# Clinical Use of Tolerogenic Dendritic Cells-Harmonization Approach in European Collaborative Effort

**DOI:** 10.1155/2015/471719

**Published:** 2015-12-24

**Authors:** Anja Ten Brinke, Catharien M. U. Hilkens, Nathalie Cools, Edward K. Geissler, James A. Hutchinson, Giovanna Lombardi, Phillip Lord, Birgit Sawitzki, Piotr Trzonkowski, S. Marieke Van Ham, Eva M. Martinez-Caceres

**Affiliations:** ^1^Department of Immunopathology, Sanquin Blood Supply, Division of Research and Landsteiner Laboratory, Academic Medical Center, University of Amsterdam, Plesmanlaan 125, 1066 CX Amsterdam, Netherlands; ^2^Institute of Cellular Medicine, Newcastle University, Newcastle NE2 4HH, UK; ^3^Laboratory of Experimental Hematology, Vaccine & Infectious Disease Institute, Faculty of Medicine and Health Sciences, University of Antwerp, Antwerp University Hospital (UZA), Wilrijkstraat 10, 2650 Edegem, Belgium; ^4^Division of Experimental Surgery, Department of Surgery, University Hospital Regensburg, Regensburg, 93053 Bavaria, Germany; ^5^Division of Transplantation Immunology & Mucosal Biology, MRC Centre for Transplantation, King's College London, Guy's Hospital, London SE1 9RT, UK; ^6^School of Computing Science, Newcastle University, Newcastle NE2 4HH, UK; ^7^Institute of Medical Immunology, Charité University Medicine, Augustenburger Platz 1, 13353 Berlin, Germany; ^8^Department of Clinical Immunology and Transplantology, Medical University of Gdansk, Debinki 7, 80-952 Gdansk, Poland; ^9^Immunology Division, Germans Trias i Pujol University Hospital, Department of Cellular Biology, Physiology, and Immunology, Universitat Autònoma Barcelona, Campus Can Ruti, 08916 Barcelona, Spain

## Abstract

The number of patients with autoimmune diseases and severe allergies and recipients of transplants increases worldwide. Currently, these patients require lifelong administration of immunomodulatory drugs. Often, these drugs are expensive and show immediate or late-occurring severe side effects. Treatment would be greatly improved by targeting the cause of autoimmunity, that is, loss of tolerance to self-antigens. Accumulating knowledge on immune mechanisms has led to the development of tolerogenic dendritic cells (tolDC), with the specific objective to restrain unwanted immune reactions in the long term. The first clinical trials with tolDC have recently been conducted and more tolDC trials are underway. Although the safety trials have been encouraging, many questions relating to tolDC, for example, cell-manufacturing protocols, administration route, amount and frequency, or mechanism of action, remain to be answered. Aiming to join efforts in translating tolDC and other tolerogenic cellular products (e.g., Tregs and macrophages) to the clinic, a European COST (European Cooperation in Science and Technology) network has been initiated—A FACTT (action to focus and accelerate cell-based tolerance-inducing therapies). A FACTT aims to minimize overlap and maximize comparison of tolDC approaches through establishment of minimum information models and consensus monitoring parameters, ensuring that progress will be in an efficient, safe, and cost-effective way.

## 1. The Case for Cell-Based Therapy in Autoimmunity, Allergy, and Transplantation

The healthy immune system is well balanced to protect against invading harmful pathogens or cancerous cells, whilst maintaining a state of unresponsiveness (“tolerance”) to our self-tissues and harmless substances [1]. Breakdown of immunological tolerance can lead to unwanted, detrimental reactions that cause autoimmune diseases (AID) like rheumatoid arthritis (RA), type 1 diabetes (T1D), or multiple sclerosis (MS) and allergies such as allergic asthma and food allergies. These immune-mediated diseases are a major disease burden. Worldwide, it is estimated that almost 1 in 10 individuals (7.6%–9.4%) [2] suffer from AID, and 1 in 9 have a recorded diagnosis of allergy.

Rejection of allogeneic tissues and graft-versus-host disease (GvHD) are unwanted immune reactions that present major barriers to successful solid organ and bone marrow transplantation. Many factors influence reactivity to foreign transplants, the most fundamental ones of which are graft antigenicity and the contribution of alloreactive effector T cells [3]. Unravelling the rules of donor-recipient histocompatibility has enabled tens of thousands of tissue, organ, and stem cell transplantations to be performed in Europe annually [4]. Nevertheless, as perfect matching of tissue-type is not usually possible, most transplant recipients depend upon lifelong generalised immunosuppression that primarily targets T cells to prevent transplant rejection or GvHD [5].

Existing therapies to treat or prevent AID, allergy, and transplantation reactions mostly include chronic treatment with immunomodulatory drugs. These drugs however are not curative and are inevitably associated with a risk of immediate or late-occurring severe adverse effects (e.g., life-threatening infections, cancer). In addition, general immunosuppressive therapy may become ineffective over time as the physiology of the patient changes (e.g., when neutralising antibodies are induced against a biological agent), low-grade immune reactions ensue, or the pathological mechanisms of disease change under continuous therapeutic pressure. Application and continued monitoring of these lifelong therapies represent an enormous economic burden for society and have a dramatic impact on the quality of patients' lives. Hence, there is an unmet need for more effective and safer therapies aimed at inducing or restoring immune tolerance [6].

**Figure 1:**
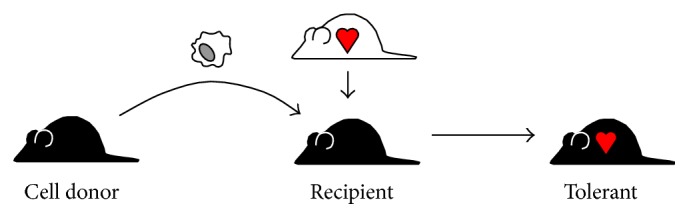
Adoptive transfer of immunoregulatory function. Transplantation of cells with immunoregulatory function to control unwanted immune reactions is not a new proposition. From the earliest discovery that transferring regulatory cells from tolerant to nontolerant animals could establish tolerance in the recipient, it was suggested that the same principle could be applied therapeutically in man. However, while adoptive transfer became a common experimental practice, its translation to the clinic met many obstacles, not least the difficulty of identifying and isolating human regulatory cells.

The principle of adoptive transfer of immunological function with purified populations of leucocytes has long been known to experimental immunologists (Figure 1). From the very earliest discovery of transferrable suppressor cell populations in animals, it was proposed that cell transplantation could be used as a tolerance-promoting therapy in humans [7]. Recent scientific and technological advances have enabled the identification, isolation, and* ex vivo* manipulation of various types for use as therapeutic agents. The development of cell-based therapies is clinically attractive for many reasons, not least the prospect of low-toxicity and antigen-specific therapies. More remarkably, because immunological tolerance is a self-reinforcing state [8], the therapeutic effects of cell therapy can outlive the therapeutic cells themselves, opening the possibility of curative treatments. Several cell types are now in early-stage clinical trials as adjunct immunosuppressive agents, including various types of regulatory T cells (Tregs) [9] or tolerogenic antigen-presenting cells (including tolerogenic DC (tolDC) and regulatory macrophages (Mregs)) [10–13]. At the present time, it is unclear which of these cell types will prove most suitable as a cell-based therapy; indeed, each has its particular advantages. Here, we describe the collaborative efforts of the A FACTT consortium to tackle the scientific, clinical, and regulatory obstacles to the implementation of therapy with tolAPC.

## 2. Mononuclear Phagocytes and the Maintenance of Peripheral Tolerance

Precisely to avoid the autoimmune and hypersensitivity reactions described above, immunological responses must be controlled at many levels. During their development, T cells, B cells, and NK cells undergo selective processes that limit their potential for self-reactivity; however, this “central” tolerance alone does not fully account for nonresponsiveness to self and innocuous foreign antigens. Many cooperating mechanisms of “peripheral” tolerance have now been described, including peripheral clonal deletion, anergy, exhaustion, deviation, ignorance, and regulation. In the last 15 years, the preeminent role of active, cell-mediated regulation has emerged from studies of regulatory cell populations, most notably FoxP3^+^ Tregs. Subsequently, the dependence of T cell-mediated regulation on tolAPC [14] became a subject of intense research. It is now firmly established that specialised subpopulations of mononuclear phagocytes are indispensable for the induction and maintenance of self-tolerance [15], as well as preventing constitutive inflammation in response to nonpathological stimuli [16].

**Figure 2:**
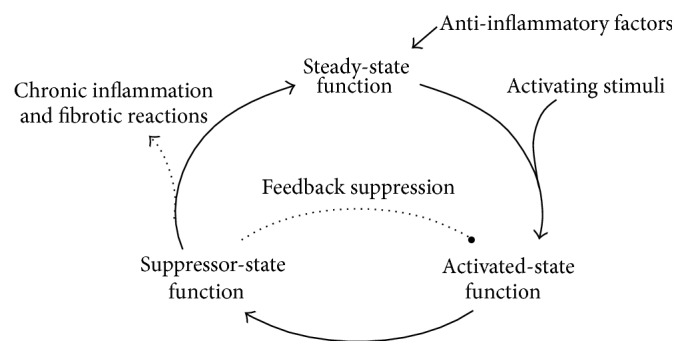
Mononuclear phagocytes are vital for control of inflammatory responses. Mononuclear phagocytes are highly adaptable effector cells that engage in diverse, often antagonistic processes: DC and macrophages are capable of both stimulating or suppressing T cell-mediated responses depending upon their state of activation. Under normal physiological, noninflammatory conditions, immature DC and macrophages present self and innocuous antigens to T cells in a subimmunogenic context. Recognition of cognate antigen in the absence of costimulation causes effector T cells to die, become anergic, or convert into regulatory T cells. Thereby, antigen presentation by nonactivated mononuclear phagocytes contributes to the steady-state maintenance of self-tolerance. A second “class” of myeloid regulatory cell arises as a consequence of persistent stimulation with proinflammatory mediators. Such activation-induced myeloid suppressor cells presumably serve as counterregulators that limit self-injurious inflammatory responses. Activation-induced myeloid regulatory cells are phenotypically diverse and operate through a variety of mechanisms, including production of T cell-suppressive soluble factors, receptor-mediated killing of effector T cells, and the activation-dependent induction of Tregs.

Tolerogenic function is not limited to any particular subset of mononuclear phagocytes; more confusingly, different regulatory DC and macrophages subsets can act through similar cellular and molecular mechanisms. Reflecting on the role of mononuclear phagocytes in the cycle of orderly inflammation may help to explain this apparent redundancy (Figure 2). Macrophages and DC are normal constituents of tissue stroma, serving vital functions in maintaining tissue homeostasis by eliminating necrotic cells and suppressing inflammatory responses against innocuous stimuli. Under steady-state noninflammatory conditions, tissue-resident DC also migrate to lymphoid tissues via afferent lymphatics where they contribute to the maintenance of peripheral T cell tolerance of self and other nonharmful antigens. Macrophages and DC in tissues are exquisitely sensitive to pathogenic signals from their environment, which drive their maturation to an immunogenic state. Activation of mononuclear phagocytes in tissues initiates the acute inflammatory cascade, including further recruitment of inflammatory monocytes from blood, often resulting in secondary tissue injury. Activated DC rapidly migrate into lymphoid tissues to stimulate adaptive immune responses, a key property of inflammatory DC. Importantly, the acute inflammatory reaction is generally self-limiting, which is partly due to repetitively and intensely stimulated mononuclear phagocytes switching to an anti-inflammatory mode. Hence, macrophages and DC can show suppressor functions both as immature cells and as poststimulatory antigen-presenting cells.

## 3. tolDC and Mregs as Therapeutic Cell Product to Restore Tolerance

**Figure 3:**
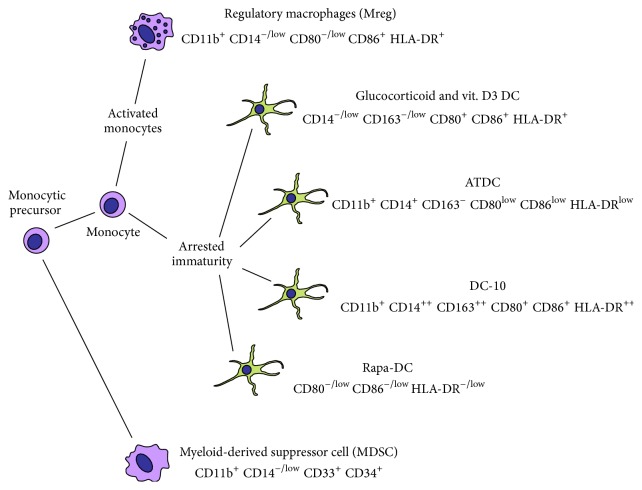
tolAPC types being developed as immunosuppressive cell-based medicinal products. The spectrum of myeloid regulatory cell products currently being developed as medicinal products is diverse, so it is valuable to categorise them as cells in arrested states of immaturity (tolDC), activation-induced suppressor cells, or myeloid-derived suppressor cells. Examples of different tolDC products are depicted.

The essential role of mononuclear phagocytes in the induction and maintenance of transplant tolerance, especially the many demonstrations that this activity could be adoptively transferred with purified DC or macrophage populations, spurred great interest in the prospect of using tolAPC to suppress pathogenic immune responses [17, 18]. Given the phenotypic plasticity of mononuclear phagocytes, it is perhaps unsurprising that a wide selection of alternative monocyte-derived cell types has been developed as potentially therapeutic cell types [19]. Most attention has focused on treating DC to drive them into a state of arrested immaturity; however, other groups are currently developing therapeutic cell products based on poststimulatory monocyte-derived suppressor cell types or myeloid-derived suppressor cells from early monocyte progenitors (Figure 3).

While the “default” function of DC is to induce tolerance, activated DC have the ability to promote destructive T cell responses. Hence it is clear that maintaining DC in activation-resistant state is an absolute prerequisite for tolDC therapy. tolDC can be defined as a maturation-resistant cell with an immature or semimature phenotype (e.g., low expression of costimulatory molecules) and stable prominent expression of anti-inflammatory molecules and low expression of proinflammatory cytokines. In order to achieve this, several biological and pharmacological agents have been evaluated to generate tolDC* in vitro* [20–25]. Since nuclear translocation of the nuclear factor kappa-light chain-enhancer of activated B cells (NF-*κ*B) is one of the major cellular processes following stimulation with a proinflammatory mediator, several agents that block this pathway and consequently the maturation process of DC have been tested to generate tolDC* in vitro*, including the NF-*κ*B inhibitor, Bay11-7082, vitamin D_3,_ dexamethasone, or IL-10 [26–28]. In addition vitamin D_3_ has also been demonstrated to interfere with cellular metabolism. It counteracts the metabolic shift towards higher glycolysis and progressive loss of mitochondrial oxidative phosphorylation following inflammatory activation [29]. Furthermore, DC can be modified genetically by knocking out immunogenic functions or by inserting tolerogenic characteristics. Overall, tolDC generated* in vitro* using these agents have been demonstrated to reduce symptoms of established AID or to prevent the rejection of transplanted tissues in experimental animal models [30, 31]. These promising outcomes have been instrumental in the development of tolDC therapy for the treatment of human AID and prevention of transplant rejection. Hereunto, a number of methods to generate tolDC* in vitro* have been translated according to Good Manufacturing Practice (GMP) for clinical use in the last 15 years.

Another clinically advanced example of an activation-induced monocyte-derived suppressor cell is Mreg [32]. Through their adherence to plastic surfaces, exposure to serum components, and stimulation with IFN-*γ*, monocytes are matured to suppressive macrophages that act through indoleamine 2,3-dioxygenase- (IDO-) dependent mechanisms [13]. Mregs express CD86 and HLA-DR, as well as high levels of other maturation-associated markers, like CD274. At least* in vitro*, human Mregs are capable of deleting activated T cells, suppressing T cell proliferation, and driving naïve T cells to become induced Tregs [33].

## 4. tolAPC-Based Clinical Trials

Several preparations of tolAPC have been tested in phase I clinical trials. Trials with autologous tolDC have been completed for T1D (USA) [12], RA (Australia [10], UK, and South Korea [34]) and Crohn's disease (Spain) [11] (Table 1). So far the results are highly encouraging from a safety standpoint, since none of the trials have found any major concerns that will prevent further testing. tolDC therapy was well tolerated by the patients, and, importantly, autoimmunity in treated patients was not enhanced. Further phase I tolDC trials are underway in MS (Spain, Belgium, and Russia), neuromyelitis optica (Spain), T1D (The Netherlands), and kidney transplantation (France). Furthermore, phase II trial with tolDC in T1D patients (USA) will start to recruit patients imminently. Mregs containing cell products have now been administered as an adjunct immunosuppressive therapy to more than 20 kidney transplant recipients with promising early results [13, 35, 36]. This therapeutic approach is now being extended in the One Study [37].

## 5. Collaborative Efforts to Overcome Limitations in tolAPC Therapy

As summarized above, several tolAPC products have been or are being tested in clinical trials. Due to the manufacturing and regulatory complexities associated with initiating a cellular therapy, relatively few groups are preparing or conducting trials with cell-based tolerance-inducing therapies (CTT) in Europe or worldwide. Specific meetings or forums are lacking, since most scientists attend disease-specific meetings or general immunology meetings in which the CTT field, including tolAPC, is underrepresented. Research groups working in kidney transplantation recently initiated a joint initiative in CTT (“One Study” EU consortium), aiming to evaluate CTT in living-donor kidney transplantation; alternatively, many other groups working in other types of organ transplantation or AID are developing their projects independently. Due to this widely distributed and limited action in CTT, joint action is needed to integrate experiences, to share results, and to discuss the strategies to go forward with clinical applications of new clinical trials. To achieve this the EU COST consortium A FACTT (action to focus and accelerate cell-based tolerance-inducing therapies) was initiated in 2014 to accelerate the development and implementation of all CTT, including tolDC and Mreg, by creating a forum for the exchange and integration of knowledge and expertise. This is the first European initiative to bring together different disciplines in the context of human immune tolerance with the main objective to accelerate and advance the clinical application of CTT treatment of AID, allergy, and prevention of graft rejection.

Regarding tolAPC current limitations of this therapy are related to both the production process and evaluation of the clinical trials, which are intended to provide information for the postulated mechanism(s) of action. The first steps to be undertaken by the A FACTT consortium will help move the tolDC field forward by addressing key issues in a collaborative effort between different labs and interests. The most important ones of these issues are discussed below.

### 5.1. Comparison of tolDC Production Protocols

One outstanding issue within the cellular therapies field is the variation in the methods used for extraction, production, and use of cells for therapeutic purposes. Different methodologies make it difficult to directly compare different cell products, therefore bringing uncertainty when ultimately comparing final efficacy and safety results. One solution to this problem is to define a set of standard protocols; however, this approach would be difficult since it would require substantial changes to existing methods from many laboratories. As part of the A FACTT project, we are defining a less radical approach of providing a standard reporting framework. We call this MITAP (Minimum Information about Tolerogenic Antigen-Presenting Cells). These guidelines make differences and similarities of approaches immediately clear and transparent. We believe that this approach has a much higher chance of being used by the CTT community as it also provides a checklist for authors when, for example, describing their methods in papers; MITAP makes their jobs easier rather than adding to the burden of scientific publication. We have tested MITAP within the A FACTT community and are ready to release the final version within the immediate future.

### 5.2. Consensus on Functional Quality Control Parameters

In general, clinically applicable tolDC can be defined as a maturation-resistant cell with MHC II expression and an immature or semimature phenotype (low or limited expression of CD80, CD83, and CD86) and stable prominent expression of anti-inflammatory membrane molecules and/or secreted products and low expression of proinflammatory cytokines, even in presence of environmental proinflammatory signals. Another limitation is that there is no consensus on how to determine the “tolerogenicity” (potency) of the tolDC product, given that tolerance can be achieved by different mechanisms, hereby restricting standardisation of functional quality control (QC) parameter(s), rendering the comparison between products in terms of functionality and safety between different laboratories difficult.

Often functional assays such as a suppression assay or an allostimulatory capacity assay are considered as potency assay. However it has to be taken into consideration that this type of assays is slow and not very precise. Therefore the use of suitable “surrogate” potency markers has to be regarded, for example, the release of inhibitory molecules (IL-10, TGF*β*, and IDO), the surface expression of certain surface markers, or even the lack of certain molecules. For this insight into the tolDC products and their mechanism of tolerance induction is important.

Understanding the fundamental biological relationships between alternative tolDC products is a key objective of the A FACTT consortium. Appreciating the similarities and dissimilarities between cell types and how these differences dictate the pharmacological properties of those cells as therapeutic agents is critical to the efficient advancement of the field. Via A FACTT we aim to discuss and share experience to create a consensus and position on a minimal set of functional QC parameters, again documented using the above-mentioned MITAP, hereby making it possible in the future to compare different tolDC approaches.

### 5.3. Harmonization of Immunomonitoring

Interpretation of the results obtained from immunomonitoring of tolAPC trials is a difficult task due to the variety of methods and protocols available to detect specific T cell responses. The lack of harmonized immunomonitoring protocols for analysis of treated patients makes it difficult to compare outcome of individual trials, decelerating the potential progress of the field.

The capability of tolAPC therapy to suppress pathogenic T cell responses* in vivo* needs to be monitored before and after administration of the tolerance-inducing cell products to determine the effects of tolAPC therapy on the immune system and to correlate these effects with clinical outcomes. Limitations in harmonization of immunomonitoring are due to limited insight into* in vivo* mechanisms of tolerance and lack of proven biomarkers. A FACTT aims to create a consensus and position on relevant immunomonitoring assays and will emphasize the use of minimal information models to describe them. To achieve harmonization for the performance of specific flow-cytometric and functional assays, standardised methods, panels, and sampling conditions will be recommended through publications and focused workshops.

### 5.4. Regulations

tolAPC are substantially modified cells and therefore must be classified in Europe as somatic cell advanced therapy medicinal products (ATMP). This has been imposed by the Regulation (EC) number 1394/2007 of the European Parliament and of the Council [38]. The most important consequence of this approach is that ATMP are treated similarly to other biological medicinal products and not as cells. Marketing authorisation approval (MAA) for such products is centralized via European Medicinal Agency and the path to offer ATMP to the patients is substantially longer when compared to cells for transplantation or transfusion/blood products, as they must be checked in a series of preclinical tests and in subsequent expensive clinical trials similarly to other classes of drugs. In some cases, this path is difficult to achieve as the cells cannot be defined to the level possible for small-molecule or even biological drugs. Although this is recognized by regulatory bodies, it adds to already very high standards of GMP required to produce cells for clinical use. Since 2007, when regulations were introduced, only five ATMP hold centralized MAA (none of them tolDC) in Europe, which illustrates difficultness of the regulations. Elusive promise of financial reward and very specific expertise necessary to develop ATMP distracts big pharma from investing in this branch of medicine and therefore academic hospitals, universities, and small-sized enterprises (usually academia-based) with limited resources are still the main manufacturers of ATMP. For obvious economic reasons, the regulations create significant hurdles for such organizations and significantly delay the translation of tolDC application.

A FACTT aims to streamline the interaction with the regulatory authorities, in which the opinion and experience of leading groups in CTT are represented, via discussions with authorities and via position papers. Hereby, A FACTT aims to create awareness that therapeutic cells have different mechanisms of action and a different safety profile compared to conventional chemical drugs and thus need unconventional regulatory requirements [39]. Furthermore by sharing preclinical data necessary for the Investigational Medicinal Product Dossier, A FACTT aims to avoid effort duplication for preclinical studies.

## 6. Conclusion

Overall, by creating a forum for researchers and clinicians working in the field of CTT therapy, experiences should be shared to enable upcoming trials based on the experience gained in previous trials. This approach saves money in duplicating work and will likely optimize outcomes for future trials. Expertise from ongoing or completed tolDC trials will be shared by our partners with laboratories preparing for new CTT (e.g., through short-term scientific missions). We envisage that the A FACTT collaborative effort will be an important step to accelerate the implementation of CTT in the clinic.

## Figures and Tables

**Table 1:** Completed phase 1 safety studies using tolerance-inducing DC.

Group	Indication	Cell culture conditions	Antigen (Ag)	Treatment regimen	Route of administration	Outcomes	Ref.
Giannoukakis, Trucco Pittsburgh, USA	Type 1 diabetes	Use of antisense ODN targeting CD40, CD80, and CD86 in mo-DC	No Ag	4 injections of 1 × 10^7^ cells every two weeks	Intradermal	(i) No AE (ii) No clinical effect (iii) Treatment-associated increase of B220+ CD11c+ B cells (iv) Evidence for reactivation of C-peptide in subjects that were C-peptide negative	[12]

Thomas Brisbane, Australia	RA	Addition of Bay11-7082 to mo-DC cultures	Citrullinated peptides: collagen type II_1237–1249_-Cit1240, fibrinogen *α* chain_717–725_-Cit720, fibrinogen *β* chain_433–441_-Cit436, and vimentin_447–455_-Cit450	1 injection of low-dose (0.5–1 × 10^6^ cells) or high-dose (2–4.5 × 10^6^ cells)	Intradermal	(i) Grade 1 AE (injection site reactions, transient leucopenia, and headache) (ii) No induction of disease flares and reduction of DAS28 in treated patients (iii) Systemic anti-inflammatory effect based on CRP levels, reduced frequency of Teff, and proinflammatory cytokines and chemokines in treated patients	[10]

Panes, Benitez-Ribas, and Ricart Barcelona, Spain	Crohn's disease	Addition of dexamethasone and vitamin A to mo-DC cultures	No Ag	Dose-escalation study: a single or 3 consecutive injections at 2-week intervals of 2 × 10^6^, 5 × 10^6^, and 10 × 10^6^ cells	Intraperitoneal	(i) No AE (3 patients withdrew because of worsening of disease symptoms) (ii) Clinical improvement in 3 out of 13 patients (1 clinical remission, 2 clinical responses) (iii) Increase of circulating Treg and decrease in IFN-*γ* levels	[11]

Hilkens, Isaacs Newcastle upon Tyne, UK	Inflammatory arthritis	Addition of dexamethasone and vitamin D to mo-DC cultures	Autologous synovial fluid	Dose-escalation study: a single injection of 1 × 10^6^, 3 × 10^6^, and 10 × 10^6^ cells	Intra-articular	(i) No evidence of acute toxicity (ii) Treatment acceptable to patients	

Joo, Bae Seoul, South Korea	RA	CreaVax-RA (autologous tolerogenic DC)	recombinant PAD4, RA33, citrullinated-filaggrin and vimentin	5 injections of low-dose (0.5 × 10^7^ cells) and high-dose (1.5 × 10^7^)	Not indicated	(i) Treatment was well tolerated (ii) Antigen-specific autoantibodies decreased in 5/9 autoantibody-pos. patients	[34]

## Abbreviations

A FACTT: Action to focus and accelerate cell-based tolerance-inducing therapies
ATMP: Advanced therapy medicinal products
AID: Autoimmune diseases
COST: European Cooperation in Science and Technology
CTT: Cell-based tolerance-inducing therapies
DAMP: Danger associated molecular patterns
GMP: Good Manufacturing Practice
GvHD: Graft-versus-host disease
MAA: Marketing authorisation approval
MITAP: Minimum Information about Tolerogenic Antigen-Presenting Cells
Mreg: Regulatory macrophage
MS: Multiple sclerosis
NF-*κ*B: Kappa-light chain-enhancer of activated B cells
PAMP: Pathogen associated molecular patterns
QC: Quality control
RA: Rheumatoid arthritis
tolAPC: Tolerogenic antigen-presenting cells
tolDC: Tolerogenic dendritic cells
T1D: Type 1 diabetes.
